# Multifaceted Effects of L-Cysteine, L-Ascorbic Acid, and Their Derivatives on the Viability and Melanin Synthesis of B16/F10 Cells under Different Conditions

**DOI:** 10.3390/antiox13030330

**Published:** 2024-03-07

**Authors:** Joon Yong Choi, Jae Won Ha, Yong Chool Boo

**Affiliations:** 1 Department of Biomedical Science, The Graduate School, Kyungpook National University, 680 Gukchaebosang-ro, Jung-gu, Daegu 41944, Republic of Korea; halo134679@knu.ac.kr (J.Y.C.); jaewon1226@knu.ac.kr (J.W.H.); 2 BK21 Plus KNU Biomedical Convergence Program, Kyungpook National University, 680 Gukchaebosang-ro, Jung-gu, Daegu 41944, Republic of Korea; 3 Department of Molecular Medicine, School of Medicine, Kyungpook National University, 680 Gukchaebosang-ro, Jung-gu, Daegu 41944, Republic of Korea; 4 Cell and Matrix Research Institute, Kyungpook National University, 680 Gukchaebosang-ro, Jung-gu, Daegu 41944, Republic of Korea

**Keywords:** L-cysteine, L-ascorbic acid, L-cysteinamide, tyrosinase, L-tyrosine, L-3,4-dihydroxyphenylalanine, alpha-melanocyte-stimulating hormone, hyperpigmentation, hypopigmentation

## Abstract

The total melanin synthesis in the skin depends on various melanogenic factors, including the number of viable melanocytes, the level of melanogenic enzymes per cell, and the reaction rate of the enzymes. The purpose of this study is to examine the effects of L-cysteine (L-Cys), L-ascorbic acid (L-AA), and their derivatives on the tyrosinase (TYR) activity and autoxidation of L-3,4-dihydroxyphenylalanine (L-DOPA) in vitro and the viability and melanin synthesis of B16/F10 cells under different conditions. L-Cysteinamide (C-NH_2_), glutathione (GSH), L-Cys, L-AA, and *N*-acetyl L-cysteine (NAC) inhibited the catalytic activity of TYR in vitro. L-AA, C-NH_2_, L-ascorbic acid 2-*O*-glucoside (AAG), and 3-*O*-ethyl L-ascorbic acid (EAA) inhibited the autoxidation of L-DOPA in vitro. L-DOPA exhibited cytotoxicity at 0.1 mM and higher concentrations, whereas L-tyrosine (L-Tyr) did not affect cell viability up to 3 mM. L-AA, magnesium L-ascorbyl 2-phosphate (MAP), and L-Cys attenuated the cell death induced by L-DOPA. C-NH_2_ decreased the intracellular melanin level at the basal state, whereas L-AA, MAP, and AAG conversely increased it. C-NH_2_ reduced the number of darkly pigmented cells via in situ L-DOPA staining, whereas L-AA, MAP, GSH, and AAG increased it. C-NH_2_ decreased the intracellular melanin level at the alpha-melanocyte-stimulating hormone (α-MSH)-stimulated state, while NAC and GSH increased it. L-AA and C-NH_2_ decreased the intracellular melanin level at the L-Tyr-stimulated state, but NAC and GSH increased it. L-Ascorbyl tetraisopalmitate (ATI) showed no or minor effects in most experiments. This study suggests that L-AA can either promote or inhibit the different melanogenic factors, and C-NH_2_ can inhibit the multiple melanogenic factors consistently. This study highlights the multifaceted properties of L-Cys, L-AA, and their derivatives that can direct their therapeutic applications in hyperpigmentation, hypopigmentation, or both disorders.

## 1. Introduction

Melanin pigment, a major factor in determining skin color, is synthesized in melanocytes located at the interface between the dermis and epidermis and distributed to keratinocytes throughout the epidermis [1,2]. Melanin synthesis in melanocytes is stimulated by various internal and external factors and regulated by multiple signaling pathways [3]. Excessive accumulation or deficiency of melanin in any skin area beyond the normal range of melanin levels of surrounding skin throughout the body can cause hyperpigmentation and hypopigmentation disorders, respectively [4].

The microphthalmia-associated transcription factor (MITF) plays a central role in the phenotypic expression of melanocytes and regulates the expression of enzymes involved in melanin synthesis, such as tyrosinase (TYR), tyrosinase-related protein 1 (TYRP1), and dopachrome tautomerase (DCT, TYRP2) [5]. In the melanosome organelle, TYR initiates melanin synthesis, and the monophenolase and diphenolase activities of this enzyme oxidize L-tyrosine (L-Tyr) and L-3,4-dihydroxyphenylalanine (L-DOPA) to dopaquinone [6]. When dopaquinone is spontaneously converted to leukodopachrome and oxidized to dopachrome, it enters the eumelanin synthesis pathway, and when dopaquinone reacts with L-cysteine (L-Cys) to generate conjugates, it enters the pheomelanin synthesis pathway [7]. Skin color depends on the relative level and distribution of pheomelanin, which is a yellow-red pigment, and eumelanin, which is a brown-black pigment [8].

L-Cys has been shown to inhibit the catalytic activity of TYR in vitro [9] and bind to the copper ion in the active site of TYR to inactivate it [10]. The effects of various thiol compounds on TYR enzyme activity, cellular melanin synthesis, and skin pigmentation vary, depending on the individual compound [11]. In our study, L-cysteinamide (C-NH_2_) inhibited TYR-mediated dopachrome formation in vitro and eumelanin synthesis in cells more effectively than L-Cys, *N*-acetyl L-cysteine (NAC), or glutathione (GSH) [12]. C-NH_2_ was considered to inhibit eumelanin synthesis by diverting dopaquinone to the formation of pheomelanin or by inactivating TYR through copper chelation activity [12].

L-Ascorbic acid (L-AA, vitamin C) can inactivate TYR in vitro by an oxygen-dependent mechanism, probably because its oxidation produces hydrogen peroxide that is removable by catalase [13]. L-AA at high concentrations inhibits TYR activity, reducing the production of dopaquinone, but at the same time causes L-DOPA accumulation, and after all L-AA is consumed, melanin synthesis can abruptly increase [14]. L-AA can rather promote melanin production in cells under certain conditions [15]. Currently, there is no strong evidence supporting the efficacy of L-AA as a skin depigmenting agent [16]. Nevertheless, various types of derivatives of L-AA, such as magnesium L-ascorbyl 2-phosphate (MAP) [17], L-ascorbic acid 2-*O*-glucoside (AAG) [18], 3-*O*-ethyl L-ascorbic acid (EAA) [19], and L-ascorbyl tetraisopalmitate (ATI) [20], have been developed and tested as skin depigmenting or anti-wrinkle agents.

Oxidative stress, which is accompanied by the excessive production of reactive oxygen species (ROS) and the depletion of antioxidants, can exert varied effects on the fate and function of melanocytes, depending on its severity [21,22]. For example, UV rays and airborne fine dust can induce oxidative stress and stimulate melanin synthesis, causing hyperpigmentation [23,24]. Severe oxidative stress can cause irreversible damage to cell components, such as nucleic acids, proteins, and lipids, and the death of melanocytes, which are associated with the development of hypopigmentation disorders, such as vitiligo [25,26]. Therefore, certain types of antioxidants may be useful in attenuating hyperpigmentation or hypopigmentation.

The total amount of melanin synthesis in the skin depends on various melanogenic factors, including the number of viable melanocytes, the melanogenic capacity of the cells, and the kinetics of melanogenic enzymes [11,26,27]. Previous studies reported the effects of L-Cys, L-AA, and their derivatives on only one or two melanogenic factors [12,13,15,28], and some results were contradictory, so there were limitations in determining their applicability in the treatment of hyperpigmentation or hypopigmentation. The present study was undertaken to gain comprehensive insight into the therapeutic applicability of L-Cys, L-AA, and their derivatives by comparatively examining their effects on the catalytic activity of TYR, the autoxidation of L-DOPA in vitro, and the viability and melanin synthesis of murine melanoma B16/F10 cells under the basal and stimulated conditions. As a result, we observed that each test compound exhibited varied effects on different melanogenic factors and identified several compounds potentially applicable to hyperpigmentation, hypopigmentation, or both disorders.

## 2. Materials and Methods

### 2.1. Reagents

L-Cysteine (L-Cys), *N*-acetyl L-cysteine (NAC), glutathione (GSH), L-ascorbic acid (L-AA), L-ascorbic acid 2-*O*-glucoside (AAG), L-tyrosine (L-Tyr), L-3,4-dihydroxyphenylalanine (L-DOPA), 2′,7′-Dichlorodihydrofluorescein diacetate (DCFH-DA), and alpha-melanocyte-stimulating hormone (α-MSH) were purchased from Sigma-Aldrich (St. Louis, MO, USA). L-Cysteinamide (C-NH_2_) was purchased from Watanabe Chemical Ind., Ltd. (Hiroshima, Japan). Magnesium L-ascorbyl 2-phosphate (MAP) and L-ascorbyl tetraisopalmitate (ATI) were purchased from Biosynth Carbosynth (Berkshire, UK). 3-*O*-ethyl L-ascorbic acid (EAA) was purchased from TCI Chemicals (Tokyo, Japan). 3-(4,5-Dimethylthiazol-2-yl)-2,5-diphenyltetrazolium bromide (MTT) was purchased from Amresco (Solon, OH, USA). Dimethyl sulfoxide (DMSO) was purchased from AppliChem GmbH (Darmstadt, Germany).

### 2.2. Assay for the Catalytic Activity of TYR In Vitro

The catalytic activity of TYR in vitro was determined by spectrophotometric measurement of dopachrome formation from L-Tyr and L-DOPA [29]. The assay mixture (200 μL) consisting of 100 mM sodium phosphate (pH 6.8), TYR enzyme preparation (20 μg protein), 1.0 mM L-Tyr, 42 μΜ L-DOPA, and a test compound at 0.1 or 0.5 mM was placed in 96-well plates (SPL Life Sciences, Pocheon, Republic of Korea) and incubated at 37 °C for up to 24 h. In this assay, a cell lysate of human embryonic kidney 293 cells constitutively expressing human TYR (HEK293-TYR) served as a TYR enzyme preparation [30]. Bio-Rad DC assay (Bio-Rad Laboratories, Hercules, CA, USA) was used to determine the protein content of the cell lysate. The absorbance of the reaction mixture was measured at 475 nm, the maximum absorption wavelength of dopachrome, using a Spectrostar Nano microplate reader (BMG LABTECH GmbH, Ortenberg, Germany).

### 2.3. Assay for L-DOPA Autoxidation In Vitro

A spectrophotometric method was used to monitor the autoxidation of L-DOPA to dopachrome [31]. A reaction mixture (200 μL) containing 1.0 mM L-DOPA and a test compound at 0.1 or 0.5 mM in phosphate-buffered saline (PBS) was placed in 96-well plates and incubated at 37 °C for up to 24 h. The oxidation of L-DOPA to dopachrome was measured by the absorbance at 475 nm using a microplate reader.

### 2.4. Cell Culture

Murine melanoma B16/F10 cells (ATCC CRL-6475) were obtained from the American Type Culture Collection (Manassas, VA, USA) and cultured in a closed incubator under humidified air containing 5% CO_2_ at 37 °C. Cells were fed with a growth medium, Dulbecco’s modified Eagle medium (GIBCO-BRL, Grand Island, NY, USA), supplemented with 10% fetal bovine serum and antibiotics (100 U mL^−1^ penicillin, 100 µg mL^−1^ streptomycin, and 0.25 µg mL^−1^ amphotericin B).

### 2.5. Assay for Cell Viability

Cell viability was assessed by MTT assay [32]. Cells were seeded in 96-well plates at a density of 3.0 × 10^3^ cells per well, cultured in 200 μL of a growth medium for 24 h, and then treated with indicated concentrations of test compounds for 48 h. The used culture medium was discarded by suction, the adherent cells were rinsed with PBS, and 100 μL of growth medium supplemented with MTT (1.0 mg mL^−1^) was added. After maintaining the cells at 37 °C for 2 h, the medium was aspirated and discarded, and 100 μL DMSO was added to extract the formazan dye inside the cells. The absorbance of the extract at 570 nm was measured using a microplate reader.

### 2.6. Assay for Melanin Levels

A spectrophotometric method was used to quantify the intracellular and extracellular melanin levels [33]. For the assay of the basal levels of melanin in B16/F10 cells, cells were seeded in 100 mm culture dishes (SPL Life Sciences) at a density of 5.0 × 10^5^ cells per dish and cultured in 10 mL of a growth medium for 24 h, and then treated with each test compound at 0.5 mM in 10 mL of a growth medium for 72 h. For the assay of the stimulated levels of melanin in B16/F10 cells, cells were seeded in 6-well culture plates (SPL Life Sciences) at a density of 1.0 × 10^5^ cells per well and cultured in 2 mL of a growth medium for 24 h, treated with each test compound at 0.5 mM, and stimulated with 100 nM α-MSH or 3 mM L-Tyr in 2 mL of a growth medium for 72 h. In both experiments, the conditioned medium was used to measure the extracellular melanin level. The adherent cells were rinsed with cold PBS twice and lysed at 4 °C with cell lysis buffer consisting of 10 mM Tris-Cl buffer (pH 7.4), 120 mM NaCl, 25 mM KCl, 2.0 mM EGTA, 1.0 mM EDTA, 0.5% triton X-100, and a protease inhibitor cocktail (Roche, Mannheim, Germany). Following centrifugation at 14,500× *g* for 15 min at 4 °C, the supernatants were saved, and the pellet was used in measuring intracellular melanin level. Cell pellets were extracted with 130 μL of 1 N NaOH solution containing 10% DMSO at 90 °C for 30 min. The melanin levels were measured by the absorbance at 400 nm using a microplate reader.

### 2.7. In Situ L-DOPA Staining

The in situ L-DOPA staining method was used to detect cells capable of melanin synthesis [34]. B16/F10 cells were seeded in 12-well culture plates at a density of 4.0 × 10^4^ cells per well, cultured in 1 mL of a growth medium for 24 h, and then treated with indicated concentrations of test compounds for 48 h. After the conditioned medium was removed by suction, cells were fixed with 4% paraformaldehyde in PBS for 10 min and permeabilized with 0.1% triton X-100 in PBS for 2 min at 25 °C. Cells were then rinsed with PBS and incubated with 0.1% L-DOPA in PBS for 3 h at 37 °C. Cells were rinsed with PBS and cell images were captured under a phase-contrast microscope (Eclipse TS100, Nikon Instruments Inc., Melville, NY, USA).

### 2.8. Statistical Analysis

Data were analyzed using SigmaStat v.3.11 Statistical Analysis Software (Systat Software Inc., San Jose, CA, USA) and are presented as mean ± standard deviation. The presence of significant differences between groups was determined by a one-way analysis of variance at the *p* < 0.05 level. All groups were compared to each other using Duncan’s multiple range test at the *p* < 0.05 level.

## 3. Results

**Figure 1:**
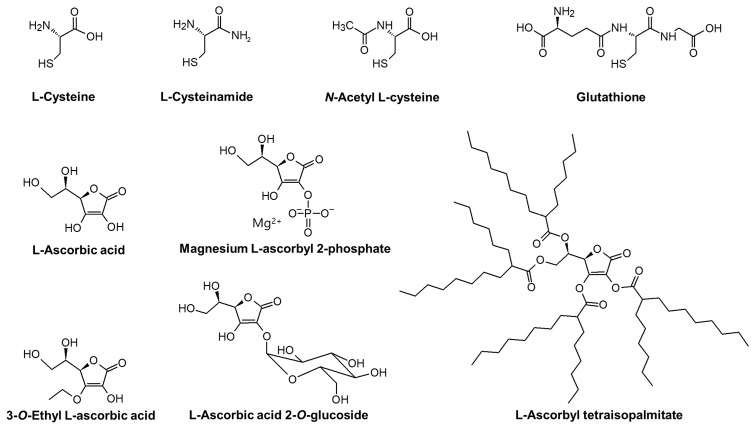
The chemical structures of L-cysteine (L-Cys), L-cysteinamide (C-NH_2_), *N*-acetyl L-cysteine (NAC), glutathione (GSH), L-ascorbic acid (L-AA), magnesium L-ascorbyl 2-phosphate (MAP), 3-*O*-ethyl L-ascorbic acid (EAA), L-ascorbic acid 2-*O*-glucoside (AAG), and L-ascorbyl tetraisopalmitate (ATI).

The chemical structures of compounds tested in the present study are shown in Figure 1.

### 3.1. Effects of L-Cys, L-AA, and Their Derivatives on the Catalytic Activity of TYR In Vitro

**Figure 2:**
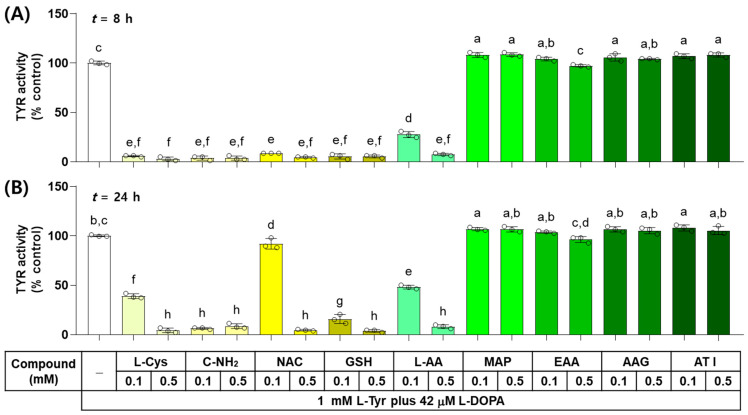
Effects of L-Cys, L-AA, and their derivatives on the catalytic activity of tyrosinase (TYR) in vitro. The reaction mixture containing TYR enzyme preparation (HEK293-TYR lysate, 20 μg protein), 1 mM L-tyrosine (L-Tyr), 42 μM L-DOPA, and each test compound at 0.1 or 0.5 mM was incubated at 37 °C for 8 h (**A**) or 24 h (**B**). TYR-mediated dopachrome formation was measured by absorbance at 475 nm. Different color bars indicate different compounds. Different lowercase letters indicate statistically significant differences between groups (*p* < 0.05).

The effects of L-Cys, L-AA, and their derivatives on the catalytic activity of TYR were examined in vitro by monitoring the dopachrome formation. As shown in Figure 2, when the reaction mixture containing each test compound at 0.1 or 0.5 mM was incubated for 8 h, L-Cys, C-NH_2_, GSH, and NAC suppressed the TYR-mediated dopachrome formation almost completely and L-AA showed a dose-dependent inhibitory effect, whereas EAA, AAG, ATI, and MAP did not exhibit inhibitory effects. When the reaction mixture was incubated for 24 h, only C-NH_2_ suppressed the TYR-mediated dopachrome formation almost completely, and the dose-dependent inhibitory effects were exhibited by GSH, L-Cys, L-AA, and NAC. The rest of the compounds did not show any inhibitory effects.

### 3.2. Effects of L-Cys, L-AA, and Their Derivatives on the Autoxidation of L-DOPA In Vitro

**Figure 3:**
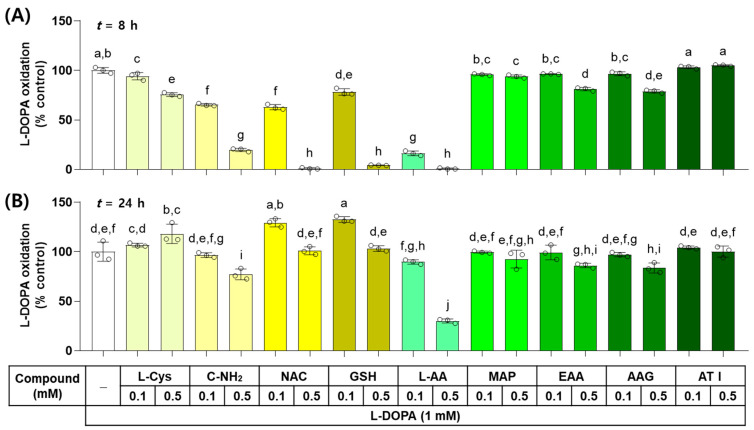
Effects of L-Cys, L-AA, and their derivatives on the autoxidation of L-3,4-dihydroxyphenylalanine (L-DOPA) in vitro. The reaction mixture containing 1.0 mM L-DOPA and each test compound at 0.1 or 0.5 mM was incubated at 37 °C for 8 h (**A**) or 24 h (**B**). The formation of dopachrome from L-DOPA was measured by absorbance at 475 nm. Different color bars indicate different compounds. Different lowercase letters indicate statistically significant differences between groups (*p* < 0.05).

The effects of L-Cys, L-AA, and their derivatives on the non-enzymatic oxidation of L-DOPA to dopachrome were examined in vitro. As shown in Figure 3, when L-DOPA (1 mM) was incubated in the presence of each test compound at 0.1 or 0.5 mM for 8 h, the dose-dependent inhibitory effects on L-DOPA autoxidation were exhibited by L-AA, NAC, GSH, C-NH_2_, L-Cys, AAG, EAA, and MAP. ATI did not show any effects. When the reaction mixture was incubated for 24 h, the dose-dependent inhibitory effects were exhibited by L-AA, C-NH_2_, AAG, and EAA in order, but GSH and NAC at 0.1 mM and L-Cys at 0.5 mM promoted L-DOPA autoxidation. The other treatments did not show any effects on it.

### 3.3. Effects of L-Tyr and L-DOPA on the Viability of B16/F10 Cells

**Figure 4:**
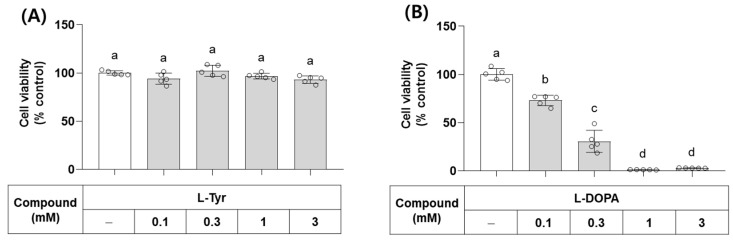
Effects of L-Tyr and L-DOPA on the viability of B16/F10 cells. Cells were treated with L-Tyr (**A**) or L-DOPA (**B**) at the specified concentrations for 48 h and subjected to cell viability assay using 3-(4,5-Dimethylthiazol-2-yl)-2,5-diphenyltetrazolium bromide (MTT). Different lowercase letters indicate statistically significant differences between groups.

L-Tyr and L-DOPA are the endogenous substrates of monophenolase and diphenolase activities of TYR, respectively. Their effects on the viability of B16/F10 cells were examined at different concentrations. As shown in Figure 4, L-Tyr did not affect cell viability up to 3 mM, but L-DOPA decreased cell viability at 0.1 mM and higher concentrations. In line with this observation, L-DOPA has been shown to cause cell death by inducing oxidative stress and/or mitochondrial dysfunction [35,36].

### 3.4. Effects of L-Cys, L-AA, and Their Derivatives on the Viability of B16/F10 Cells in the Absence and Presence of L-DOPA

**Figure 5:**
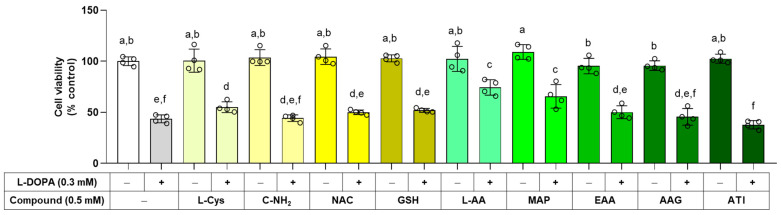
Effects of L-Cys, L-AA, and their derivatives derivatives on the viability of B16/F10 cells. Cells were treated with each test compound at 0.5 mM alone or together with 0.3 mM L-DOPA for 48 h, and cell viability was determined by MTT assay. Different color bars indicate different compounds. Different lowercase letters indicate statistically significant differences between groups.

The effects of L-Cys, L-AA, and their derivatives on cell viability were examined in the absence and presence of 0.3 mM L-DOPA. As shown in Figure 5, any of these compounds alone did not affect cell viability at 0.5 mM. L-AA, MAP, and L-Cys attenuated cell death induced by external L-DOPA, and the rest of the compounds did not affect it.

### 3.5. Effects of L-Cys, L-AA, and Their Derivatives on the Basal Melanin Levels of B16/F10 Cells under a Normal Culture Condition

**Figure 6:**
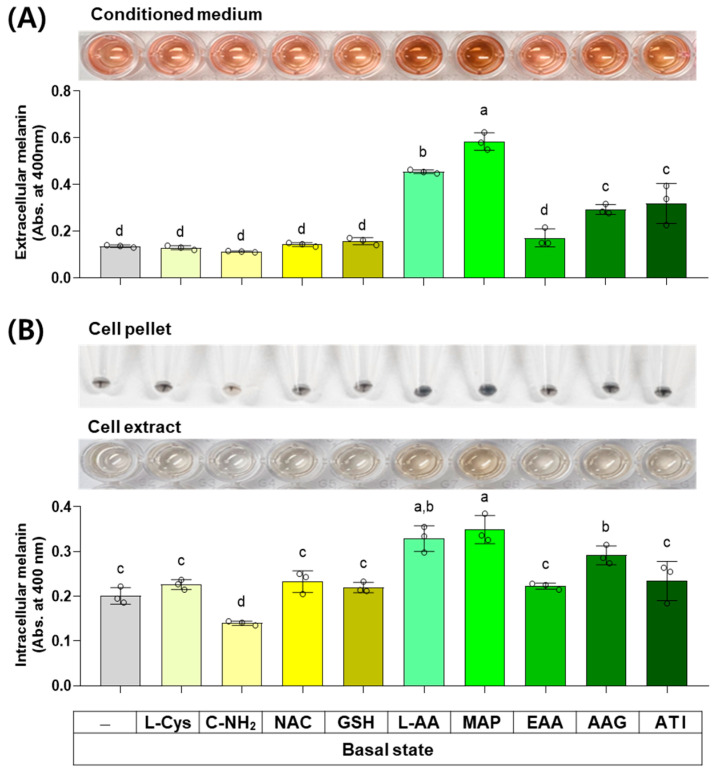
Effects of L-Cys, L-AA, and their derivatives on the basal melanin levels of B16/F10 cells under a normal culture condition. Cells grown in 100 mm culture dishes were treated with each test compound at 0.5 mM for 48 h. The conditioned media and adherent cells were harvested and used for the measurement of the extracellular (**A**) and intracellular melanin levels (**B**), respectively, by absorbance at 400 nm. The images of conditioned media, cell pellets, and cell extracts are shown. Different color bars indicate different compounds. Different lowercase letters indicate statistically significant differences between groups (*p* < 0.05).

The next experiment compared the effects of L-Cys, L-AA, and their derivatives at 0.5 mM on the basal melanin levels of B16/F10 cells under a normal culture condition. As shown in Figure 6, MAP, L-AA, ATI, and AAG increased the extracellular melanin level at the basal state, and other compounds did not affect it. C-NH_2_ decreased the intracellular melanin level, whereas L-AA, MAP, and AAG conversely increased it. Other compounds did not affect the intracellular melanin level.

### 3.6. Effects of L-Cys, L-AA, and Their Derivatives on the Number of Darkly Pigmented Cells via In Situ L-DOPA Staining

**Figure 7:**
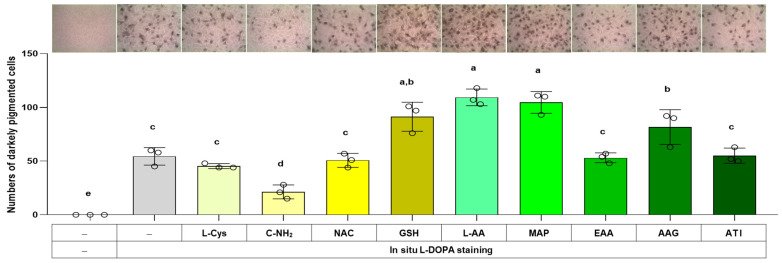
Effects of L-Cys, L-AA, and their derivatives on the melanin synthesis capacity of B16/F10 cells assessed by in situ L-DOPA staining. Cells grown in 6-well culture plates were treated with each test compound at 0.5 mM for 48 h and then subjected to in situ L-DOPA staining. The images of L-DOPA-stained or control cells are shown. Different color bars indicate different compounds. Different lowercase letters indicate statistically significant differences between groups (*p* < 0.05).

The total melanin level in a cell is related to the product of the cell’s melanin synthesis capacity (i.e., the level of melanogenic enzymes) and the melanin synthesis rate (i.e., the rate of reaction catalyzed by melanogenic enzymes). The effects of L-Cys, L-AA, and their derivatives on the cell’s melanin synthesis capacity were examined in the following experiment using the in situ L-DOPA staining method. Cells were treated with different compounds for 48 h and then fixed and subjected to L-DOPA staining. As shown in Figure 7, C-NH_2_ reduced the number of darkly pigmented cells, whereas L-AA, MAP, GSH, and AAG augmented it. This indicates that cellular melanin synthesis capacity was downregulated by C-NH_2_ and upregulated by L-AA, MAP, GSH, and AAG.

### 3.7. Effects of L-Cys, L-AA, and Their Derivatives on the Melanin Levels of B16/F10 Cells Stimulated by α-MSH

**Figure 8:**
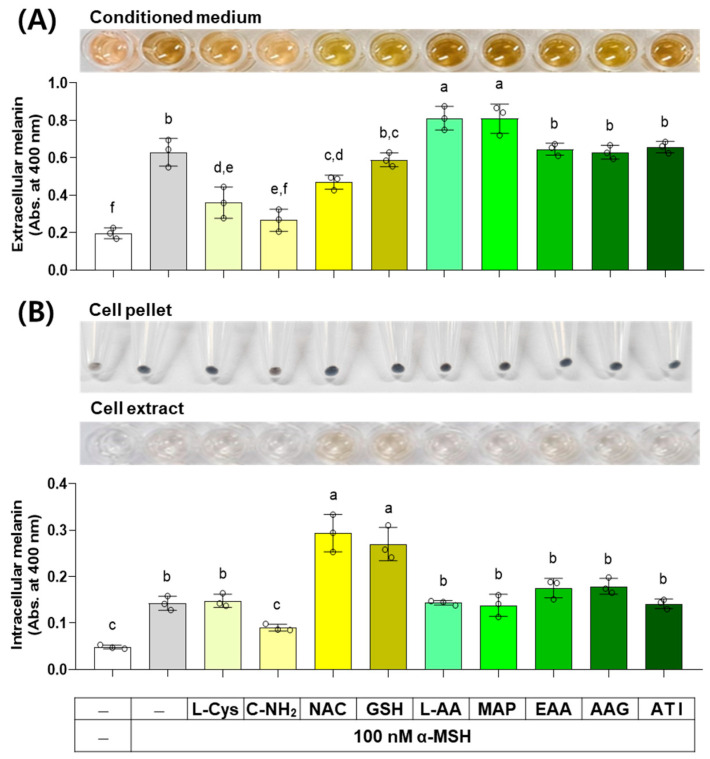
Effects of L-Cys, L-AA, and their derivatives on the melanin levels of B16/F10 cells stimulated by alpha-melanocyte-stimulating hormone (α-MSH). Cells grown in 6-well culture plates were treated with each test compound at 0.5 mM and 100 nM α-MSH for 48 h. The conditioned media and adherent cells were used for the measurement of the extracellular (**A**) and intracellular melanin levels (**B**), respectively, by absorbance at 400 nm. The images of conditioned media, cell pellets, and cell extracts are shown. Different color bars indicate different compounds. Different lowercase letters indicate statistically significant differences between groups (*p* < 0.05).

α-MSH activates the cAMP response element-binding protein (CREB) transcription factor through a protein kinase A (PKA)-dependent mechanism, and the subsequent expression and activation of the MITF transcription factor induce the expression of TYR, TYRP1, and DCT, thereby increasing melanin synthesis capacity [37]. The results of an experiment investigating the effect of several compounds on melanin levels in cells stimulated by α-MSH are shown in Figure 8. C-NH_2_, L-Cys, and NAC decreased the extracellular melanin level at the α-MSH-stimulated state, while L-AA and MAP increased it, and other compounds did not affect it. C-NH_2_ decreased the intracellular melanin level at the α-MSH-stimulated state, while NAC and GSH increased it, and other compounds did not affect it.

### 3.8. Effects of L-Cys, L-AA, and Their Derivatives on the Melanin Levels of B16/F10 Cells Stimulated by L-Tyr

**Figure 9:**
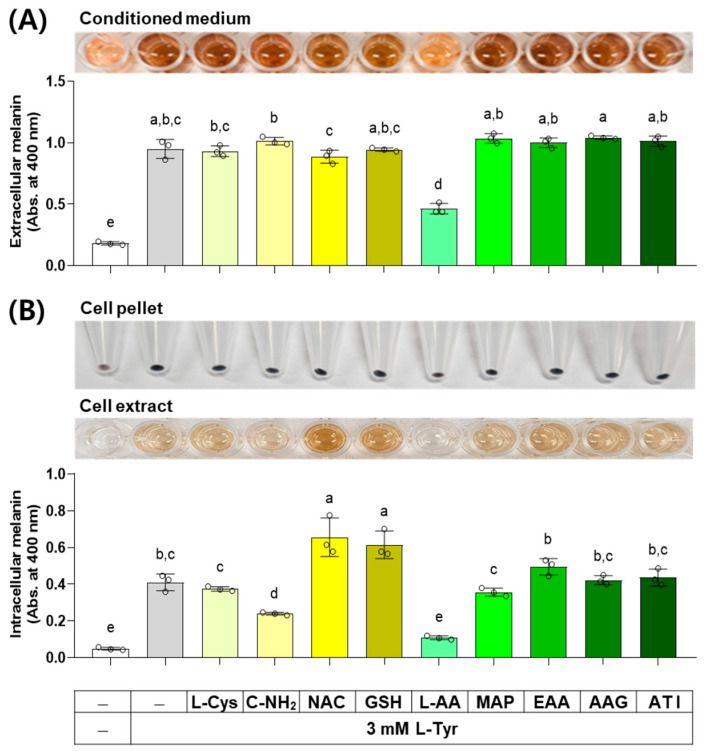
Effects of L-Cys, L-AA, and their derivatives on the melanin levels of B16/F10 cells stimulated by L-Tyr. Cells grown in 6-well culture plates were treated with each test compound at 0.5 mM and 3 mM L-Tyr for 48 h. The conditioned media and adherent cells were used for the measurement of the extracellular (**A**) and intracellular melanin levels (**B**), respectively, by absorbance at 400 nm. The images of conditioned media, cell pellets, and cell extracts are shown. Different color bars indicate different compounds. Different lowercase letters indicate statistically significant differences between groups (*p* < 0.05).

When external L-Tyr is supplied to cells at a high concentration, the melanin synthesis by melanogenic enzymes in cells increases [38]. The results of an experiment investigating the effect of several compounds on melanin levels in cells stimulated by L-Tyr are shown in Figure 9. L-AA decreased the extracellular melanin level at the L-Tyr-stimulated state, whereas other compounds did not affect it. L-AA and C-NH_2_ decreased the intracellular melanin level at the L-Tyr-stimulated state, NAC and GSH enhanced it, and other compounds did not affect it.

## 4. Discussion

The present study revealed the multifaceted effects of L-Cys, L-AA, and their derivatives on multiple melanogenic factors through the measurement of various parameters under different experimental conditions, as summarized in Table 1.

The basal level of intracellular melanin was very low compared to the α-MSH- or L-Tyr-stimulated level, and a more concentrated cell extract prepared from more cells was required for quantitative analysis. To meet this requirement, a higher number of cells were cultured and treated in 100 mm culture dishes in an experiment to measure the basal levels of melanin, whereas the cells were cultured and treated in six-well culture plates in other experiments to measure the stimulated levels of melanin. In addition, some compounds caused different quantitative changes in extracellular and intracellular melanin levels. Assuming that extracellular melanin measurements may be more affected by experimental artifacts, data discussion prioritized intracellular melanin levels. Some compounds slightly increased the activity of TYR or the autoxidation of L-DOPA in vitro, as measured by absorbance at 475 nm. The reasons for these unexpected results are unclear. Unless the results were affected by simple experimental error(s), the compounds or their oxidized forms may have promoted the production of dopachrome by acting as prooxidants or increased the measured absorbance or optical density by forming unknown chromophores or complexes with other components of the reaction mixture.

L-AA is a water-soluble antioxidant and an essential cofactor of a group of 2-oxoglutarate-dependent dioxygenases, including prolyl 4-hydroxylase, prolyl 3-hydroxylase, and lysyl 5-hydroxylase, which mediate the post-translational modification of procollagen proteins [39]. There is consensus that L-AA may help prevent skin aging associated with collagen loss [40]. Meanwhile, the effects of L-AA on melanin synthesis and skin hyperpigmentation remain inconclusive [16]. The present study showed that L-AA has different effects on several melanogenic factors. L-AA increased the viability of cells exposed to a cytotoxic level of L-DOPA, the intracellular melanin level at the basal state, and the number of darkly pigmented cells via in situ L-DOPA staining. On the other hand, L-AA inhibited the catalytic activity of TYR and L-DOPA autoxidation in vitro and decreased the intracellular melanin level at the L-Tyr-stimulated state. The results indicate that L-AA has distinct properties that can attenuate both hyperpigmentation and hypopigmentation of the skin.

Because L-AA is an unstable polar compound, there are limitations in using it in cosmetics or medicines and applying it directly to the skin [40]. Studies have reported the cosmeceutical or therapeutic utility of various L-AA derivatives developed to enhance physicochemical stability [17,18,41], increase hydrophobicity and skin absorption [19,42,43,44], or impart multi-functionality [45]. Most L-AA derivatives are supposed to be converted to L-AA in biological systems. In the present study, MAP and AAG, which are hydrophilic L-AA derivatives, increased the intracellular melanin level at the basal state and the number of darkly pigmented cells via in situ L-DOPA staining, as L-AA did. However, EAA and ATI, which are lipophilic L-AA derivatives, did not show any remarkable effects in cells. The route of their administration via aqueous medium and the rate of their biochemical conversion to L-AA might have limited the action of these lipophilic L-AA derivatives.

L-Cys can act as an antioxidant because of a sulfhydryl group. In the present study, L-Cys inhibited the catalytic activity of TYR and the autoxidation of L-DOPA in vitro, and it increased the viability of cells exposed to a cytotoxic level of L-DOPA. However, L-Cys did not affect the intracellular melanin level at the basal state nor the number of darkly pigmented cells via in situ L-DOPA staining. In addition, it did not affect the intracellular melanin level at the α-MSH- and L-Tyr-stimulated states either.

NAC is a derivative of L-Cys developed to improve absorption through the cell membranes or the skin [46]. In the present study, NAC inhibited the catalytic activity of TYR and the autoxidation of L-DOPA in vitro but did not affect the intracellular melanin level at the basal state nor the number of darkly pigmented cells via in situ L-DOPA staining. Unexpectedly, NAC slightly increased the intracellular melanin level at both the α-MSH- and L-Tyr-stimulated states.

GSH is a tripeptide that plays an important role in redox homeostasis and antioxidant defense [47,48]. When supplied from outside, GSH cannot be directly absorbed into cells but must be metabolized into free amino acids that can enter the cell by specific transporters [49]. Thus, it is difficult to define the mechanism of action of external GSH, whether it is direct or indirect, although several clinical studies have reported that administration of GSH or glutathione disulfide (GSSG) in different routes alleviates melasma and hyperpigmentation [11]. In the present study, GSH inhibited the catalytic activity of TYR and the autoxidation of L-DOPA in vitro but had no significant effects on the intracellular melanin level at the basal state. However, it increased the number of darkly pigmented cells via in situ L-DOPA staining and the intracellular melanin levels at both the α-MSH- and L-Tyr-stimulated states.

In our previous study, among 20 amidated amino acids, only C-NH_2_ inhibited the catalytic activity of TYR in vitro and melanin synthesis in human melanoma MNT1 cells and normal human epidermal melanocytes [12]. In the present study, C-NH_2_ inhibited the catalytic activity of TYR and the autoxidation of L-DOPA in vitro. In addition, it consistently reduced the intracellular melanin level at the basal state, the α-MSH-stimulated state, and the L-Tyr-stimulated state. Its effects on the viability of B16/F10 cells exposed to a cytotoxic level of L-DOPA were not significant.

Several thiol compounds, such as L-Cys, C-NH_2_, NAC, and GSH, can react with dopaquinone during melanin synthesis and switch from eumelanin synthesis to pheomelanin synthesis [11,12,38]. Despite this common property, the thiol compounds were found to exhibit different and, in some cases, opposing effects on the total melanin content of cells in the present study. Therefore, it is suggested that these thiol compounds may inhibit or promote overall melanin synthesis by influencing various melanogenic factors in cells, regardless of their effect on switching eumelanin synthesis to pheomelanin synthesis. The melanin analysis method in this study, which measures absorbance at 400 nm, can detect both eumelanin and pheomelanin with different sensitivities [50], so it cannot be ruled out that a shift between eumelanin and pheomelanin by thiol compounds may have affected the measured melanin content. This possibility needs to be further examined using other methods, but considering that eumelanin is the predominant form of melanin pigment in B16/F10 cells [51], the impact of the changed pheomelanin content on the measured total melanin content is unlikely to be significantly large.

Overall, the observations with L-Cys, NAC, and GSH in the present study do not support their alleviating effects on hyperpigmentation but rather raise the possibility that NAC and GSH may conversely enhance pigmentation. In contrast, the unique property of C-NH_2_ consistently inhibiting multiple melanogenic factors in vitro and cells under different conditions supports its therapeutic potential in attenuating skin hyperpigmentation.

## 5. Conclusions

**Figure 10:**
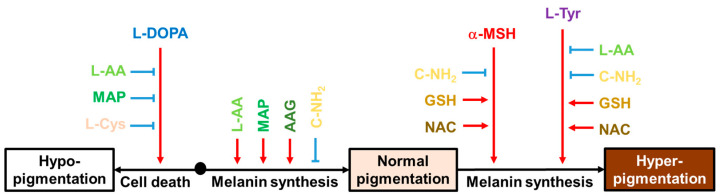
Effects of various compounds on the cell death induced by L-DOPA and the intracellular melanin levels at the basal state, α-MSH-stimulated state, and L-Tyr-stimulated state. Black arrows indicate the cellular events associated with different pigmentation levels of the skin. Red arrows indicate stimulation or promotion and blue blunted arrows indicate inhibition or suppression by external hormones or compounds (different color letters).

This study shed new light on the multifaceted properties of L-Cys, L-AA, and their derivatives that affect multiple melanogenic factors at the cellular level, as schematized in Figure 10. In particular, L-AA and C-NH_2_ showed notable effects in cells under different conditions. L-AA rescued cells from L-DOPA-induced death and increased the intracellular melanin level at the basal state while decreasing the intracellular melanin level at the L-Tyr-stimulated state. C-NH_2_ consistently decreased intracellular melanin levels at the basal state and both the α-MSH- and L-Tyr-stimulated states without affecting cell viability. This study suggests that certain antioxidants could be used in attenuating hyperpigmentation, hypopigmentation, or both disorders. Further, in vivo experiments and clinical trials are warranted for the therapeutic application of these compounds based on the findings from this study.

## Figures and Tables

**Table 1:** Effects of various compounds on the catalytic activity of TYR and L-DOPA autoxidation in vitro and the viability and melanin levels of B16/F10 cells under different conditions. ↑, ↓, and = represent increases, decreases, and no changes, respectively (*p* < 0.05).

Measurement Parameters	Compounds (0.5 mM)								
	L-Cys	C-NH_2_	NAC	GSH	L-AA	MAP	EAA	AAG	ATI
TYR-mediated dopachrome formation for 8 or 24 h in vitro	↓/↓	↓/↓	↓/↓	↓/↓	↓/↓	↑/=	=/=	↑/=	↑/=
Autoxidation of L-DOPA to dopachrome for 8 or 24 h in vitro	↓/↑	↓/↓	↓/=	↓/=	↓/↓	↓/=	↓/↓	↓/↓	=/=
Viability of cells exposed to a cytotoxic level of L-DOPA	↑	=	=	=	↑	↑	=	=	=
Extracellular and intracellular melanin levels at the basal state	=/=	=/↓	=/=	=/=	↑/↑	↑/↑	=/=	↑/↑	↑/=
Number of darkly pigmented cells via in situ L-DOPA staining	=	↓	=	↑	↑	↑	=	↑	=
Extracellular and intracellular melanin levels at the α-MSH-stimulated state	↓/=	↓/↓	↓/↑	=/↑	↑/=	↑/=	=/=	=/=	=/=
Extracellular and intracellular melanin levels at the L-Tyr-stimulated state	=/=	=/↓	=/↑	=/↑	↓/↓	=/=	=/=	=/=	=/=

## Data Availability

The original contributions presented in the study are included in the article, and further inquiries can be directed to the corresponding author.
